# “I don’t want to disturb her”: a youth citizen social science community case study on the school health services in Oslo, Norway

**DOI:** 10.3389/fpubh.2026.1718850

**Published:** 2026-07-08

**Authors:** Aina Landsverk Hagen, Hilde Rønnaug Kitterød, Brita Askeland Winje, Anders Lund Hage Haugen, Turid Kristin Bigum Sundar, Ann-Karin Valle

**Affiliations:** Work Research Institute, Department of Nursing and Health Promotion, Department of Primary and Secondary Teacher Education, Oslo Metropolitan University, Oslo, Norway

**Keywords:** citizen social science, inter-professional collaboration, mental health, school health services, youth participation

## Abstract

**Introduction:**

This study explores how youth can contribute as co-researchers in the development of school health services through a Youth Citizen Social Science (YCSS) approach. Despite strong policy support for user involvement, there is limited empirical knowledge on how Norwegian school health services incorporate the perspectives of youth. This pilot study forms part of the YouthPulse pilot project and investigates how young people conceptualize mental health and experience their local school health services.

**Methods:**

A transdisciplinary research team collaborated with a junior high school in Oslo, engaging 20 students (aged 13–14) as citizen social scientists. Guided by the “House of Training” framework, the students received methodological training in interviews, surveys, and observation, and selected six subtopics related to mental health. Data were collected through peer interviews, a school-wide survey (*N* = 206), and participant observations in the class room. Findings were synthesized and presented in a Living Lab with school nurses, teachers, and municipal and national stakeholders to co-create recommendations for improving school health services.

**Results:**

Students’ findings revealed that while most pupils knew where the school health service was located, few were aware of when it was available. Barriers to access included uncertainty about confidentiality, fear of being embarrassed when talking about mental health, and lack of awareness regarding the nurse’s role. The co-research process itself enhanced students’ research literacy, ethical awareness, and sense of agency. Stakeholders emphasized that the youth-led insights illuminated communication gaps, structural barriers and the need for stronger inter-professional collaboration between schools and health services.

**Discussion:**

The pilot demonstrates that YCSS can produce meaningful knowledge on youth mental health while strengthening democratic participation and interdisciplinary learning in schools. Embedding citizen social science within curricular frameworks such as Health and Life Skills and “Efforts for others” can improve health literacy and service responsiveness. Broader implementation may foster equitable, practice-based evidence for participatory school health services nationally.

## Introduction

*Louisa came over and showed me a picture card of a pair of old hands sand explained that it was like ugly things that someone said to others, which left marks, ‘just like a piece of paper that you fold and unfold again’, there are marks left behind from what they said*.^1^ (researcher fieldnotes, May 27, 2025).

This study takes as its outset the under-researched field of youth’s own voices about the school health services. Norway’s school health services is a well-supported, universal health services for children aged 5–20 years. Norwegian guidelines emphasize that children should be heard, involved, and have influence in their contact with this service, at individual, group, and system levels (1). Despite the service’s long-standing presence and seemingly high level of political support, there is limited research on these services. Also, the extent of children’s involvement in setting priorities for these services is unclear, both in Norway and internationally.

**Figure 1:**
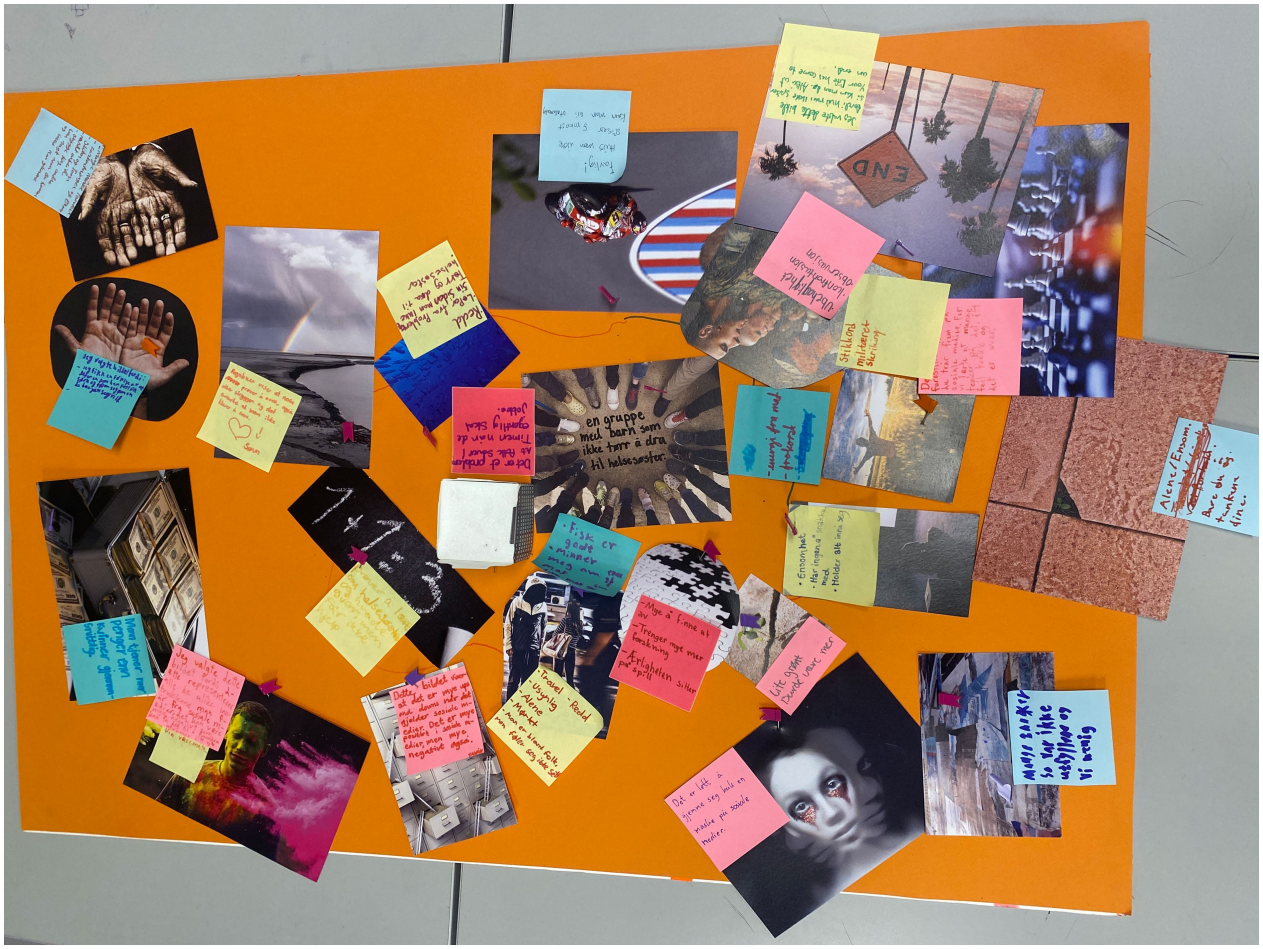
The poster with visual association cards chosen by the students to represent the “problems” about mental health among youth, based on their findings. Keywords in Norwegian. See image on top left (hands with wrinkles) chosen by Louisa with the keywords: “ugly things others have said that you cannot forget», “many dark thoughts,” “tired and empty.” The keywords on the image in the centre (a circle of children) says, “a group of children who are afraid to go to the school nurse.” This figure is adapted from the framework of (30).

The fieldnote excerpt above is from a session with young pupils in a Norwegian junior high school in the pilot project “YouthPulse.” The students were asked to find illustrative images that represented the topic of mental health of youth, a visual inquiry method we often use when engaging with youth in citizen social science interventions (2). What we call “association cards” empowers youth to put in words what is often not so easy or accessible to express (Figure 1).

Citizen social science is an emerging research approach (3) (Haklay et al., 2021), also in education (Cammarota and Fine, 2008; Solé et al., 2023), often building on long traditions of Participatory Action Research (3) (Reason and Bradbury, 2006). Additionally, the EU raised the need for more knowledge about Citizen *Social* Science (CSS) in particular, and especially about the inclusion of youth (Sis.net, 2017). What we call Youth Citizen Social Science (YCSS), can be defined as “a form of participatory social research that involves youths as citizens working together with social scientists creating and communicating new knowledge” on the social world [(4), p. 19].

When striving for youth participation and involvement in most or all aspects of the research, ethical aspects are integral to the ongoing reflective practices of both scientists and participating youth. This will be discussed below.

In previous citizen science projects conducted by the research group, mental health was emphasized by Norwegian youth, when asked in an open-ended way what was important to them (5, 6). Data from the nationwide UngData survey also shows that an increasing number of adolescents have reported mental health challenges (7). This is further reflected in school health services: in 2020, half of school nurses reported that mental health was increasingly prominent in consultations, and in 2023 most (86%) said that teaching about mental health was a main part of their work (8, 9).

This article has three aims. First, we describe the context of school health services in Norway, before we detail the community case; a pilot project in which youth co-researched pupils’ views on strategies to improve mental well-being in their local high school, focusing on school health services. Second, we discuss the youth citizen scientists’ findings on the school health services, and participants’ reflections on the process, including the teachers and the public health nurses, within the analytical framing of “scaffolding” for participation (10). Finally, we address how these findings can be applicable elsewhere and suggest pathways towards scaling such endeavours.

## School health services and inter-professional collaboration

Norwegian municipalities are legally required to provide school health services (11), supported by national guidelines to ensure consistent and equitable practice (1). In line with The UN Convention on the Right of the Child (12), these guidelines emphasize that children and adolescents should be heard and involved in interactions and decisions affecting them, both individually and systemically. Despite this clear directive, documentation of these practices is scarce.

School health services aim to promote students’ health and well-being and prevent disease in collaboration with families, schools, and the wider community (1). The services are multidisciplinary, led mainly by public health nurses (PHNs), who are employed by municipalities and not the schools. This organisational structure became visible as a barrier in our pilot study. Although collaboration between schools and health services is expected, it can be challenging in every-day work (13) and may affect service quality.

Limited resources in many municipalities also mean that public health nurses must prioritize strictly (13). Guidelines explicitly require school health services to collaborate with schools, while the Education Act places a more general obligation on schools to collaborate with other professionals, without explicitly mentioning school health services (1, 14). This difference may hinder cooperation (8, 15), even though such collaboration is both expected and works well in many settings (16).

Research shows that interprofessional collaboration can be difficult due to differences in educational backgrounds and professional cultures. Common challenges include communication and confidentiality, time constraints, leadership, contextual understanding and contact and competence in mental health (17). Our pilot study highlights an additional challenge: tensions and power dynamics that emerge when youth are actively involved in shaping the health services.

## Context

In a current renewal of the Norwegian curriculum for primary and secondary schools, the Norwegian government launched an interdisciplinary subject named Health and Life Skills (HLS). The overarching goal of the subject is to enhance pupils’ ability to make responsible decisions and promote individual and collective health and well-being. This paper’s empirical basis is a municipal case study conducted by an interdisciplinary research team including junior high school students (age 13–14) trained as youth citizen social scientists, in a public school in the Norwegian capital, Oslo during the spring of 2025. The collaboration involves the school health services in the municipal district, and public health and education scientists from the nearby university.

The purpose of the community case study was (1) to test and further develop knowledge-based methods for youth participation in research and public sector service design, (2) to capture young people’s perspectives and solutions, and ensure that they are heard and included in relevant decisions concerning public health issues and school health services development, and (3) to strengthen young people’s competence to participate in decisions that affect them, both on how they can participate and what they can expect from participation.

The pilot project took place over 1 year, spanning a spring and an autumn term at the junior high school. In both terms, researchers worked with pupils as citizen social scientists in the elective course “Efforts for Others.” This article primarily draws on the first period (February to June 2025), when about 20 8th grade pupils (aged 13–14) participated. In the autumn term the pilot continued with a 9th grade class of about 30 pupils (aged 14–15), including 10 pupils who had taken part previously; findings from this autumn period will not be discussed here.

“Effort for others” is a graded course and features well-defined learning outcomes that guide both its content and teaching methods. The two focus areas of the course are volunteering and social entrepreneurship, along with an emphasis on responsibility and collaboration (18). The pupils are intended to gain practical experience with community service activities and social responsibility in their local communities.

The aim of YouthPulse aligned well with the curriculum of the “Efforts for others” course. A primary goal of the course is for students to develop initiatives, actively participate, and utilize their own resources in ways that allow them to experience being of value to others (18). Learning aspirations include encouraging students to reflect on what contributes to an inclusive society and to follow ethical guidelines for communication with diverse target groups. In this pilot the impact of the co-research findings was to be amplified through a Living Lab session near the end of the term, where regional and national stakeholders were invited to co-create ideas with the youth based on their findings.

The pupils received scientific training based on the methodological citizen social science framework “House of Training” (4), developed by the research group at the Work Research Institute (AFI), as a result of several years of action-oriented research on youth participation. The co-research method has earlier been tested in areas such as development of youth clubs, major urban development projects, crime prevention, social inclusion and public health (2, 19, 20). The methodology developed through these past experiences of co-research was in this pilot project to be tested out, further developed and adjusted for the purpose of participation in the development of the school health services (Table 1).

**Table 1:** The table presents the roles and responsibilities of the different actors in the pilot study; students (the Youth Citizen Social Scientists), the school (teachers and administrative staff), the health services (including the school nurses) and the interdisciplinary research team.

Roles and responsibilities	Students (as YCSS)	School	Health services	Research team
Goals of youth involvement	Attend “Effort for others” class, get assessment	Teach curriculum, assess students, for students to experience impact	Get insight from youth, improve services	Test methodological framework on health service development
Research ethics and data protection	Gave informed consent, voluntary activity, anonymised interview information	Secured parents informed consent, gave informed consent in interview	Gave informed consent in interview	Ethics of care approach, integrated in YCSS framework, secured ethics approval, aggregated survey results
Defining the questions	Defined subtopics, survey and interview questions	Defined framing, learning goals	Provided information on the services to the youth	Defined main topic of mental health, school health services and youth participation
Creating the instruments/tools	Co-created the survey and interview guides, posters for dissemination	Co-created assessment tool	-	Created citizen social science framework “House of training,” visual tools (“data roll,” posters) and Living Lab design
Collecting information	Conducted the peer-interviews, informed students about the survey	Distributed survey & subjects of reflective interviews with research team	Subjects of reflective interview with research team	Set up and designed the survey based on students’ questions, participant observation in class, interviewed teacher, school nurses, school admin
Analysing information	Co-analysed collectively (using “data roll”)	Co-analysed with students	-	Co-analysed with students
Disseminating findings	Designed posters with findings and recommendations for stakeholders, leading dissemination in Living Lab	Participants in Living Lab, supporting students in communicating with stakeholders	Participants in Living Lab	Facilitated Living Lab session, supported students in communicating with stakeholders, lecturing and talks, write journal article
Roles (subject, consultant, partner, director)	Consultants, partners and directors	Consultants, partners and subjects	Subjects, consultants	Partners, directors

The table is adapted from the framework of Checkoway and Richards-Schuster (2003).

All 8th grade-students who had elected the “Effort for others” course were invited to participate in YouthPulse. If they already had filled their curriculum with other confirmed activities throughout the term, they could choose to go ahead as planned rather than participate in the pilot. An information letter was sent to all parents by the school administration, explaining the aim and content of YouthPulse, asking both parents and pupils for their consent to use the outcome of the study for research. In the case of a pupil or their parents not consenting to take part in the pilot, the pupil could still follow the same program as the rest of the class. If so, they were informed that no parts of their work, neither written or oral, would be recorded for research purposes. However, all pupils and parents consented to participate.

## Detail

At the beginning of the term, the pupils received a dual training program, facilitated by the research team. During an intensive day of training, the pupils got introduced to and practiced a range of social science methods: research interview, survey and observation. In addition, the pupils spent substantial time exploring the two central topics within their research mission: mental health and school health services. The researchers saw it as important to approach the topics in a broad and explorative way to encourage the pupils to find their own research interest within the overarching purpose of the project. This resulted in the students suggesting a variety of subtopics. Their teacher argued in an interview at the end of the term that giving the students the choice to form their own topics of interest was good for boosting their engagement.

These were the six subtopics the students decided to pursue:

1. Sleep.
2. Food.
3. Screen time and social media.
4. What youth know about mental health.
5. Mental health differences between boys and girls.
6. How the school health service at their school functions.

Further, the groups formulated research questions regarding their specific topic, prepared interview guides and survey questions with supervision from the research team. To ensure compliance with research ethics and participants’ privacy, researchers were responsible for overseeing that all research questions were targeted at systemic level and service provision level, and not on personal health information. Further, researchers guided pupils on data protection and confidentiality and instructed them to inform interviewees not to share personal information. The pupils were only allowed to make notes on paper without any name or other person identifiable information. They then independently carried out between 1 and 6 interviews per group. Most of the interviews were conducted with other pupils at their school. While pupils across the three grades (8th, 9th and 10th) were interviewed, most of the interviewees were 8th graders. The pupils also conducted interviews with the public health nurses and the school’s social educator.

**Figure 2:**
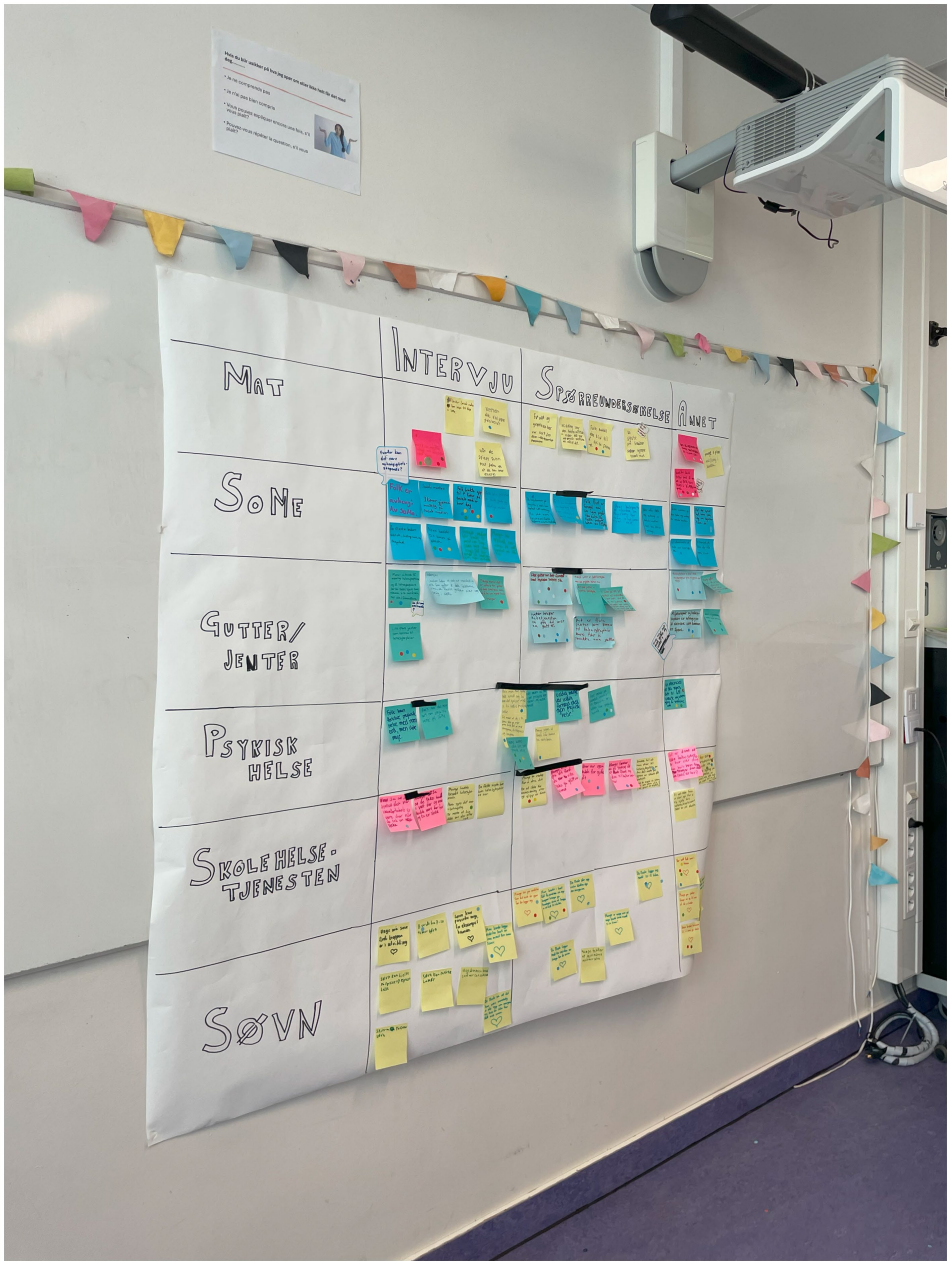
The “data roll” where the students put up their findings using post-its, to start the analysis process as a collective. The six subtopics listed above are written in the vertical column to the left, and the main research strategies (interview, survey, other) are written in the horizontal top column.

In addition to the individual thematic research interviews, each group was given the task of formulating at least three survey-questions addressing their chosen thematic focus. The researchers collected all the questions and designed a joint questionnaire, covering all six topics. This allowed researchers to ensure that no personal identifiable or sensitive questions were included. The co-created survey was sent out to the entire student body and got a 50 percent response rate (*N* = 206). The data file was assessed by researchers to prevent deductive disclosure before it was made available to the pupils. Lastly, the pupils were also encouraged to do their own observations or use information and experiences they already had regarding their topics, as background data. Additionally, the researchers “translated” some of the answers into figures and diagrams that could easily be interpreted by the students for analysing the data (Figure 2).

Throughout the analysis process, the research group introduced a variety of visual arts-based methods to facilitate the analysis (Leavy, 2015), including the association cards and poster design. The data collected through these three strategies was analysed both within and across the six thematic groups. After completing their data collection, each group was given templates designed by the researchers where they were asked to fill out what they had learned about their topic through the interviews and survey, also answering the questions “what was surprising” and “what was expected”—questions meant to prompt an incipient analysis.

The pupils then analysed the results across the six thematic focuses. A wall poster called the “data roll” gave the pupils a chance to empty their heads for all the findings and (anonymised) data they had collected through the various research approaches, share it with their peers and get an insight into the other groups’ findings. This allowed for a level of cross-thematic analysis. Further, the researchers developed a third tool to foster collective qualitative analysis: “the three Ps,” referring to findings pointing towards either something “Positive,” something “Problematic” or something regarding the “Process” (of researching).

**Figure 3:**
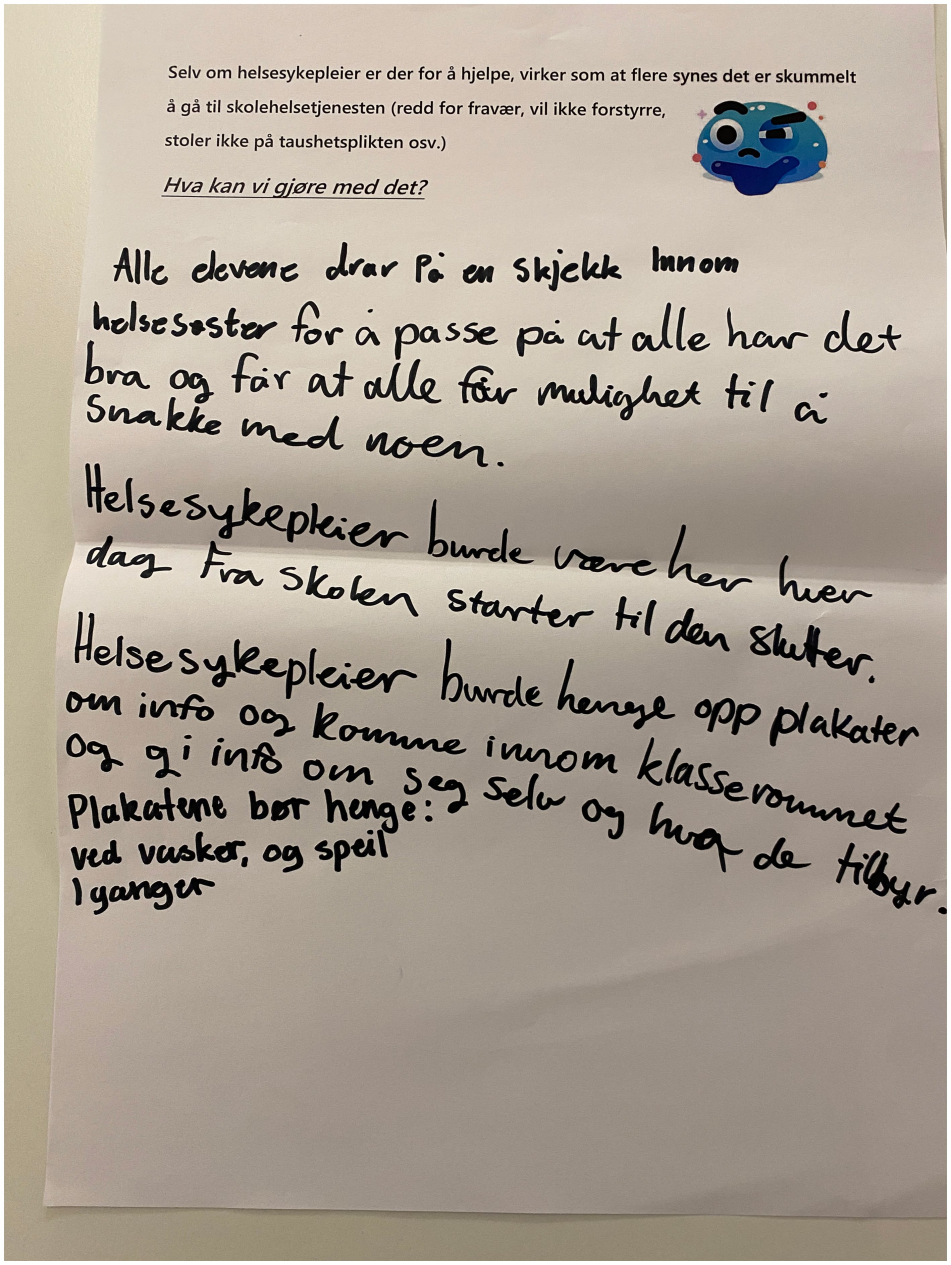
One example of the students’ notes on the first version of suggestions to the school health services, based on their findings and paradoxes (the note is written in Norwegian): “All pupils go to a check-up at the school nurse to make sure that everyone is well and gets the chance to speak with someone. The school nurse should be here every day from the school day starts until it ends. The school nurse should put up posters with information and come by all the classrooms to give information about themselves and what they offer. The posters should hang by the sinks, mirrors and in the corridor.”

Based on their data and findings, the researchers assisted the pupils in identifying “Paradoxes” in their data material. An example of such a paradox from the group researching what youth themselves know about mental health sounded as follows: *“Many report being satisfied with their own mental health. Nevertheless, it appears that several find it uncomfortable to talk about, and that many lack substantial knowledge about the topic.”* These paradoxes would in turn spark further analysis, and, most importantly, be a starting point for developing ideas and suggestions that would later be presented to adult stakeholders in the Living Lab-session at the end of term. Based on their findings the young co-researchers formulated suggestions for how the school health service could improve their work, with a particular focus on mental health. All the groups prepared a poster illustrating their research and suggestions, before presenting these at the Living Lab (Figure 3).

The participating stakeholders in the Living Lab were the public health nurses from the municipal district, a school management representative, university employed social science and health researchers, representatives from NGOs working for childrens’ rights, and a public health advisor from a municipality in the larger Oslo region. At the Living Lab, the stakeholders rotated between the group tables “hosted” by the students, who presented their findings, suggestions for improvement and the YCSS process they had been part of.

During the Lab, stakeholders were asked to take notes using a question-based template titled “Receive and translate contributions from participants.” Finally, all participants evaluated the Living Lab session anonymously. The teacher, the school management representative and the two public health nurses were interviewed about their experiences with the process and their reflections on the students’ findings by a researcher in the team just before and after the summer holiday between the two school terms.

## Findings and reflections about the outcome

**Figure 4:**
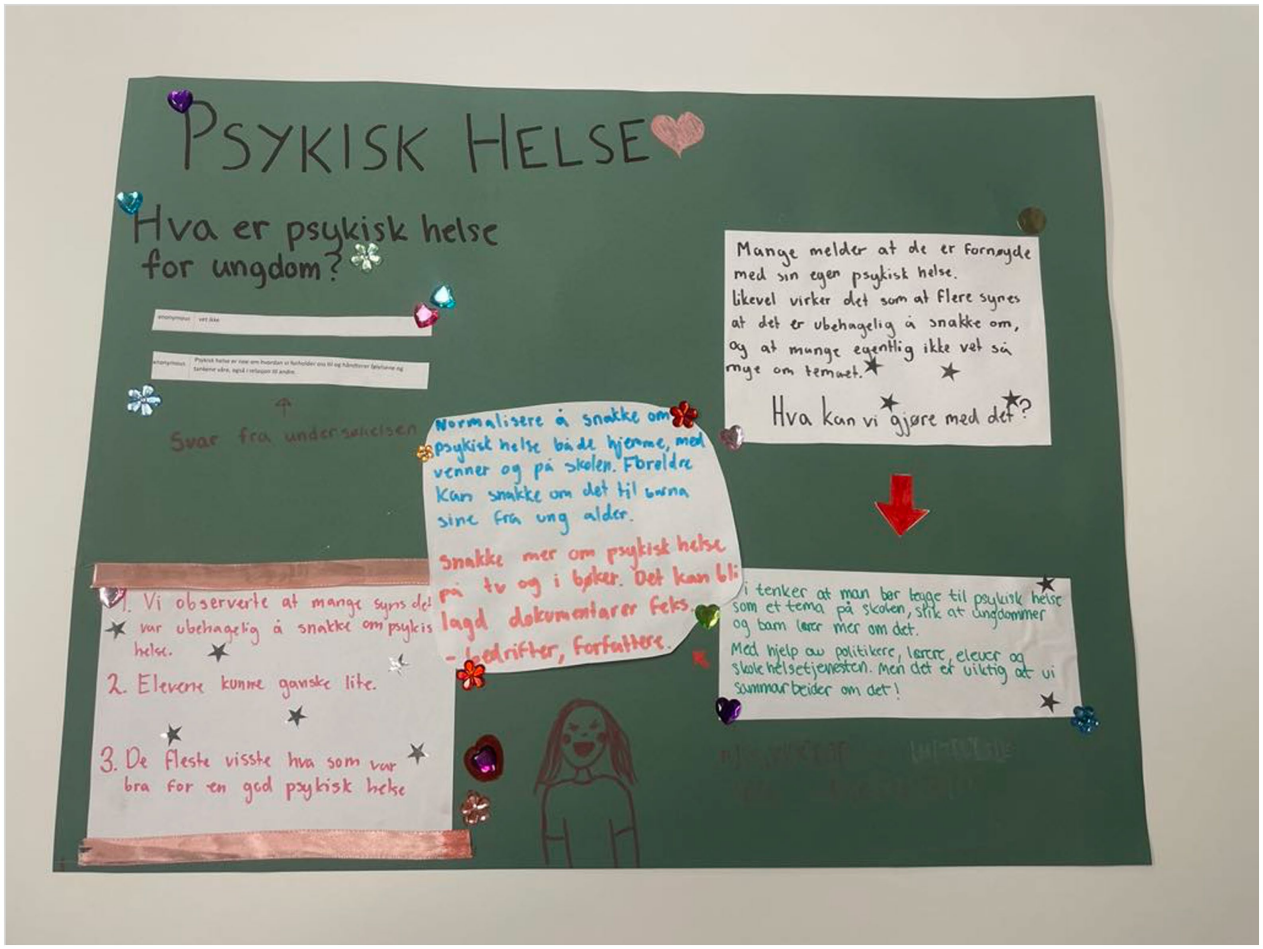
The poster summarizing the findings on mental health among youth and suggestions, presented to stakeholders at the Living Lab in June 2025. Findings summarised: “(1) We observed that many found it uncomfortable to talk about mental health. (2) The pupils knew quite little, (3) Most of them knew what was good for good mental health.” Suggestions summarised: “normalise talking about mental health both at home, with friends and at school. Parents can talk about it with their children from a young age. Talk more about mental health at TV and in books. There should be a documentary for example. We think that mental health should be taught as a topic in school so that youth and children learn more about it. With help from politicians, teachers, pupils, school health service. But it is important that we collaborate about it.”

The mental health subthemes chosen by the youth, resulted in several important findings. The survey distributed at the junior high school showed that although many pupils (age 13–16) have been in contact with the school health service, girls utilize the service more often than boys. When asked about the reason for the last contact with the school health service, one in four reported that they needed someone to talk to, were stressed or had sleeping problems. Most pupils acknowledged that physical and mental health were interconnected, and one pupil explained: “it’s very complex.” The survey showed that although most pupils knew *where* in the school building the school health service was located, only one out of five knew *when* public health nurses were available at the school (Figure 4).

**Figure 5:**
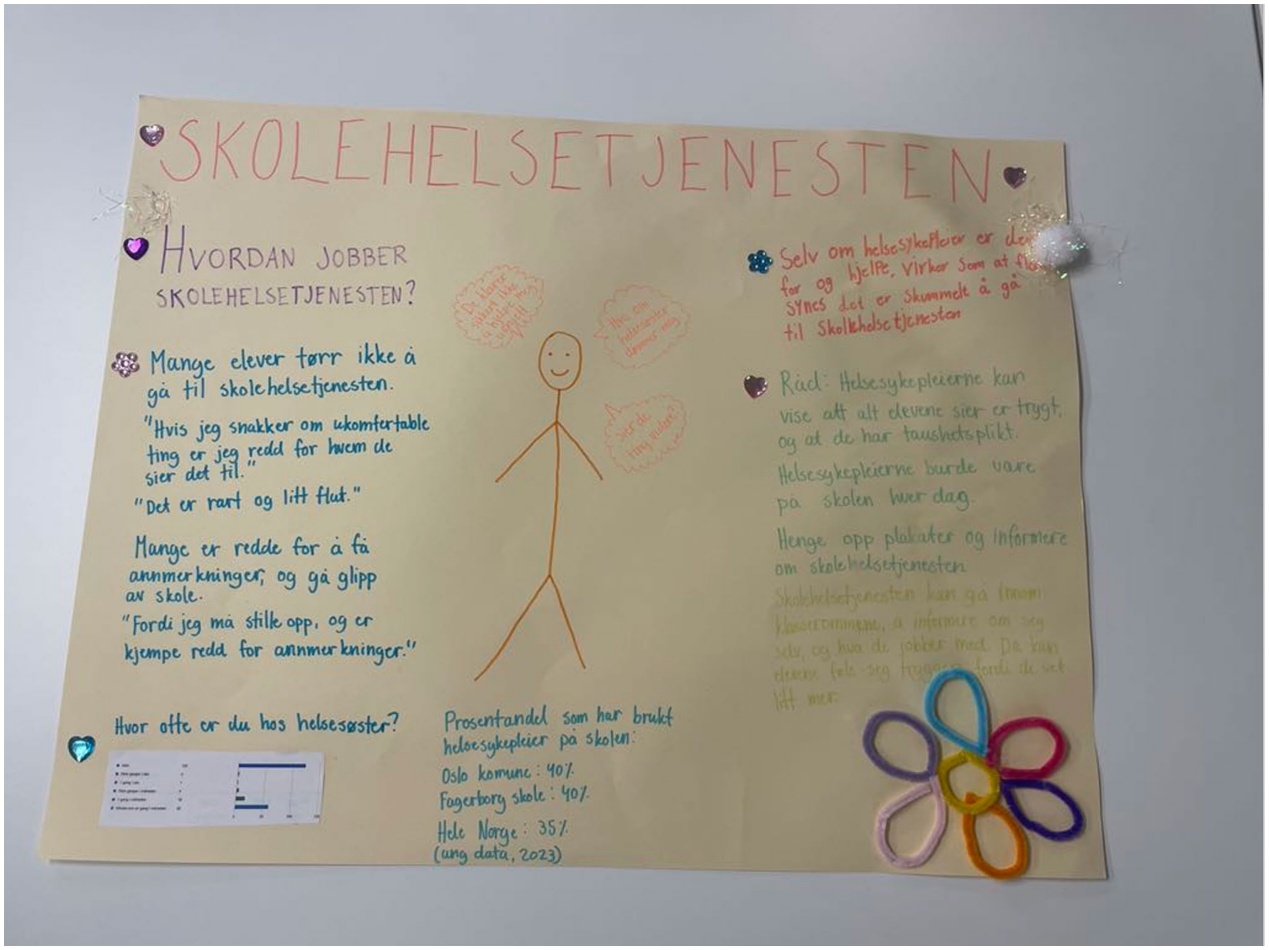
The poster summarizing the findings on the school health services and suggestions, presented to stakeholders at the Living Lab in June 2025. Findings summarised: “Many pupils are afraid of going to the school nurse. Many are scared of getting absence notes and to miss class.” Suggestions summarised: the school nurse should show the pupils that what they say is safe and confidential. The school nurse should be at school every day. Put up posters to inform about the service. The school nurse should come by the classrooms to inform about themselves and what they work with. Then the pupils might feel safer because they know more.”

The co-researchers also found that many pupils were hesitant to use the school health service and quoted from one of these interviews that “if I talk about uncomfortable things, I’m afraid of who they’ll tell.” It became clear that the duty of confidentiality is something the students struggle to understand. Also, they quoted a student saying “It’s strange and a little embarrassing” to talk to the school health nurse about mental health issues. Another finding was that many of the students are afraid of getting absence notes and of missing classes; “Because I have to show up and I’m terrified of getting absence note.” Together, we summarized another paradoxical finding: Even though the school nurse is there to help, it seems that many find it for various reasons, scary or difficult to use the school health services (Figure 5).

One pupil wrote in the survey: “I think it is difficult to understand what the school health service is.” Another wrote on the question of why he/she did not use the service more, that “I do not want to disturb her” (meaning the public health nurse).

Early on, just after their initial training, the students reflected on the tasks of interviewing: “I think I became quite good at asking questions” and “perhaps the most important thing I have learned today is how much work it takes to conduct interviews and how much time researchers spend preparing questions.” Also, they reflected on the ethics of doing social science on a potentially sensitive topic like mental health: “I have learned a lot of important things today, but one of them is to be a little careful when interviewing people.” Such feedback gave us the opportunity to discuss ethics further, next time we met the pupils. Statements like these in turn made us reflect on the scaffolding of youth participation, not only when it came to the ethics of collecting information but also on the ethics of involving the youth in such a complex, entangled web of actors (including their peers) with varying motivations. The purpose of scaffolding is to support an emerging structure until it is stable and strong enough to stand on its own. What was the scaffolding effect (or non-effect) of this pilot study, in addition to increasing the pupils’ science literacy and competences?

## Scaffolding for youth agency—contradictions and power dynamics

In the interview after the Living Lab the teacher reflected that the students “have become better at owning a process over a long period of time, at sticking with it” and “some of them have become better at taking responsibility.” Her statements were later backed by the school management representative who argued that “project-based work involves a completely different approach; pupils are active contributors.” Still, another paradox surfaced towards the end of the spring term, that the pupils reported that they learned most from listening to each other, while they were visibly afraid to speak up in plenary sessions in the classroom.

The teacher argued that “they have this project, and then they have their image to maintain.” She explained that when the Living Lab was over one student stated that ‘I have more ideas,’ but nonetheless they did not dare to say it out loud in front of their peers. Here we experienced that the choices of methods meant to shape “common ground” and minimize as far as possible the power gaps between researchers, educators, health service providers and young people, did not seem to minimize the peer pressure between the youth.

The recommendations from the pupils participating in the Living Lab, prompted the public health nurses to reflect on the youth’s perception of them. They thought it was interesting to “hear what the students thought about us,” to gain more insight into the services they provide, and exclaimed how they realized that they may need to “change the way we present ourselves.”

The public health nurses were clear in their appreciation of the suggestions from the pupils, but they also problematized the structural limitations:

“It’s very interesting to hear what they say. What they think we or the school. Or what more can be done. But then I also think that they are children who come up with these wishes. And not everything they say is achievable. So, you do not want to say ‘no, that’s not possible’. But listen to what they say and think, OK, can we meet them halfway on some things?”

From the perspective of the school, the teacher commented that “it is very good that it (the suggestions) comes from the pupils themselves, as it has more impact.” But she was also sceptical, “who is responsible for ensuring that the advice is followed?.” She argued that “the pupils must be given a reason why this is not happening” and that “they have learned a lot themselves, but there must be consequences.”

In the opinion of the public health nurses, the youth participation is already integrated in their services, since the youth come to them voluntarily and choose the topics of conversations freely. After the Living Lab they were puzzled that the pupils’ suggestions were concerning things they cover with their services, “but we do this already.” The challenge seemed to be that the pupils did not know this.

Importantly, the public health nurses explained that when they start in a position, “we find ourselves quite alone facing a large school. We would like to be welcomed into the fold. But it is up to the school how they want to include us in their activities. In their social cooperation. I feel that how the school wants to include you as a health nurse is very important.” One concrete barrier we saw in the pilot was that the public health nurses did not have access to the joint digital software and folder with the results from the students’ data collection, resulting in them not taking part of the co-analysis process.

## Discussion

We have presented a community case of a social science pilot in a junior high school in Oslo, Norway. We argue that this kind of program has the potential to not only provide valuable data on public health issues from the youth perspective, but also to bring forth urgent challenges in cross-sector, inter-professional collaboration. These are most clearly expressed by the pupils’ lack of knowledge on the availability of these services in their own school. Such insights also brought forth reflections on how the pupils on their own accord were interested in topics that were relevant to the health services, the school and the public health scientists. Additionally, despite their young age, the pupils were all reflective and capable to co-research these topics. The Living Lab participants noted that they got a valuable reminder to “remember to ask and listen to young people.”

The scaffolding effect on the learning outcomes of the school-science collaboration was clearly visible. In the Norwegian core curriculum, it is emphasized that pupils should learn through experiencing democracy in practice (18). This pilot project thus aligns closely with the national curricula by actively engaging pupils as co-researchers in developing school health services. Alignment with the curriculum is crucial for any school-based intervention, as it increases the likelihood that schools will be willing to adopt and establish similar citizen social science programs in the future.

The inter- and cross-disciplinarity involvement exposed barriers like the municipal and school employment structures, the peer pressure among youth, and their perceived limited access to and understanding of the school health services, without affording solutions within the limits of this pilot study.

## Constraints and limitations

Scaffolding youth participation in collaboration with schools with the dual aim of improving school health services and empowering students, is a complex endeavour. The pilot study has, like many citizen social science projects, conceptual and methodological shortcomings related to quality, transferability, engagement, and accessibility (21). These will be discussed below. In addition, working with youth, securing an institutional ethics of care was crucial (19, 22) but challenging, considering the nature of a small-scale pilot with limited resources to follow up the pupils’ suggestions after the pilot ended. This also affected transferability, yet one remedy here was the Living Lab design that secured direct communication between students and stakeholders. The assessment structures were not directly limiting the case study, but we struggled with keeping the youth engaged as curious and reflective co-researchers within the rather narrow school timetable.

The challenges of combining an educational infrastructure with the Public Health services agenda and the research infrastructure of seed funding, where effectiveness is subordinate to the exploratory stance of science, did take its toll – but mainly on the teachers, who struggled to integrate the pilot study with the curriculum. They explicitly asked the researchers to “translate” the activities to fit with the learning objectives of the “Efforts for others” study programme. In addition to guidance from the education researcher on the team, the adaptable framework and research principles for training, documenting and analysing results (4) helped increase quality. Also, we revised the planned design for the second term, so that the students in 9th grade would be able to test out social actions based on their own recommendations. This way we tried to mitigate the teacher’s well-funded scepticism that the pupils did not see the results of their efforts. We identify this as the main barrier when doing cross-sector participation work in collaboration with schools – who is responsible for providing feedback to the pupils, who are often dispersed after the class, term or grade has ended?

We believe this model may have wider learning outcomes and broader generalizability, particularly considering the increasing emphasis in schools on public health and life skills, interdisciplinary learning, and democracy and participation. We argue that the project design supports both the core values of the curriculum while equipping students with transferable competencies like collective problem-solving skills that are essential for navigating complex societal issues, beyond the scope of health education. The participatory design allows for multiple routes of growth, as Hart (10) argued for. As important, it can improve the services, *if* the recommendations are taken up by adult stakeholders. Again, the responsibility of giving feedback on youth’s suggestions is fragmented at best, and it seems to be one of the main structural barriers for the school to engage in similar projects in the future.

Conducting research with youth and facilitating youth participation processes always come with great ethical responsibility. For instance, the principle of voluntary participation is central in both practices. This project has followed official guidelines for processing personal data in compliance with data protection legislation in Norway, and parents provided informed consent. Still, carrying out a youth participation scheme in a school setting poses further ethical challenges, due to the obligatory nature of school attendance and the pupils’ motivation to perform well and achieve good grades. This requires a refined tuning in towards the young pupils’ sometimes non-vocal acclamations of autonomy within a school setting. Additional work is needed to understand how the young pupils may shape the agenda further, both through challenging the traditional teacher-pupil and researcher-informant hierarchies and roles, and the sector barriers that prevent co-creation throughout the process of investigating a public health topic like the youths’ mental health.

Citizen social science can provide practice-based evidence produced by practitioners about what works in their local context (21), and if scaled out to more schools and municipalities can become a knowledge base for young students, teachers and the public health services. One venue would be to create a national pupils-as-social scientists-program, to make the results from YCSS projects in collaboration with schools trustworthy, but also the processes caring, inclusive and longitudinal. Following Atal et al. (21) we argue that developing the terminology and framework of citizen social science to conduct research within and beyond educational settings, is fruitful. This will potentially provide insights, reflections and recommendations, actionable for others to test in their local school settings. The findings from this community case study do not in themselves lead to generalisable normative recommendations but can rather give inspiration to build a pool of practice-based evidence that can be tested locally by pupils, teachers and public health services. As such, we can envision participation as mutually reinforcing structures between people, not just across generations.

The push towards open science and stronger ethical commitments to non-academic interlocutors, have both shaped and encouraged our methodological explorations. The youth we call “co-researchers” or youth citizen social scientists can be seen as our “epistemic partners” (23), with many of the same functions, responsibilities and roles as traditional interlocutors. Still, there are some important differences. Further research is needed to understand how to increase the collaborative engagement of educators and practitioners within school settings, together with the youth themselves.

## Data Availability

The datasets presented in this article are not readily available because anonymization. Requests to access the datasets should be directed to haai@oslomet.no.

## Footnotes

1 All names of students are pseudonyms.
